# Intake of free sugar among children and adolescents in Germany declines – current results of the DONALD study

**DOI:** 10.1007/s00394-024-03456-1

**Published:** 2024-07-05

**Authors:** Ines Perrar, Ute Alexy, Ute Nöthlings

**Affiliations:** Institute of Nutritional and Food Sciences -Nutritional Epidemiology, University of Bonn, Friedrich-Hirzebruch- Allee 7, 53115 Bonn, Germany

**Keywords:** Free sugar, Trends, Children, Adolescents

## Abstract

**Purpose:**

Our recent analysis reported decreasing trends in intake of free sugar in children and adolescents in Germany. Here we set out to update this analysis with current dietary intake (until 2023) because of the strong public health nutrition interest in sugar intake.

**Methods:**

In total, 4,218 dietary records kept between 2010 and 2023 by 751 participants (46.0% females, 3–18 years) from the German Dortmund Nutritional and Anthropometric Longitudinally Designed (DONALD) cohort were examined. Age and time trends in free sugar intake (%E/d) were analysed using polynomial mixed-effects regression models.

**Results:**

Median intake data indicate a decline in the intake of free sugar between 2010/2011 (16.7%E) and 2022/2023 (11.7%E). Trend analyses confirmed, that intake of free sugars decreased continuously between 2017 and 2023 (Linear trend: β = -0.4126, *p* < 0.0001). In addition, free sugar intake changed significantly with age (Linear trend: β = 1.2922, *p* < 0.0001; quadratic trend: β = -0.08613, *p* = 0.0094; cubic trend: β = 0.001442, *p* = 0.1725), i.e. the intake of free sugars increases continuously up to early adolescence (9/10 years) and decreases again thereafter.

**Conclusion:**

The intake of free sugar among children and adolescents continued to decline, but still exceeded the WHO recommendations in 2023. Further measures to reduce free sugar intake would therefore be desirable, as well as continuous monitoring of sugar intake levels among this age groups.

**Supplementary Information:**

The online version contains supplementary material available at 10.1007/s00394-024-03456-1.

## Introduction

The high sugar intake of children and adolescents and its health consequences are the subject of intense debates in public health nutrition in Germany [1]. Based on observational studies on the association between free sugar intake and dental caries, the WHO recommends to limit free sugar intake to less than 10% of energy intake (%E) per day for all age groups, whereby free sugar is defined as “all monosaccharides and disaccharides added to foods by the manufacturer, cook or consumer, plus sugars naturally present in honey, syrups and fruit juices” [2]. As changes in the intake of free sugars are associated with changes in body weight, the WHO also gives the qualitative recommendation to reduce the intake of free sugars throughout the life course [2]. Therefore, the monitoring of the intake of free sugar is of public interest.

In 2020, we published detailed analyses of sugar intake based on data (1985–2016; *n* = 1312) of the Dortmund Nutritional and Anthropometric Longitudinally Designed (DONALD) study, which collects data on diet and health from infancy to adulthood since 1985 [3]. These detailed trend analyses showed among others that free sugar intake increased between 1985 and 2005 and decreased thereafter, most notably since 2010, but median free sugar intake still exceeded the WHO recommendations in the whole observation period [3]. The decline in sugar intake was also confirmed using a predictive biomarker [4].

To date, only a few studies have investigated the intake of added or free sugars among children and adolescents in Germany. Thus, there is a lack of data, especially from recent years i.e. from 2017 onwards, which would be helpful for current discussions on public health measures to reduce sugar intake. German nutrient databases generally contain data on total sugar content of the listed foods, but not on added or free sugars. As the estimation of free sugar intake requires detailed information on the nutrient composition and the estimated intake of foods, especially of commercial food products, the calculation is not possible using standard food composition databases. In the DONALD study the continuously updated in-house nutrient database LEBTAB [5] is used, which includes data on brand-specific sugar content of commercial products as well as sugars or sweetening agents such as syrups and honey, which are used for food preparation at home. This data can be used for the estimation of added and free sugar intake. Therefore, the aim of this analysis is (1) to describe current data on sugar intake of children and adolescents and (2) to examine most recent trends in free sugar intake based on data of the German DONALD study.

## Methods

### Study design and population

The DONALD study is a dynamic cohort study collecting data on diet, growth, development and metabolism of healthy children and adolescents since 1985 in Dortmund, Germany. Every year, 35–40 infants are newly recruited. Eligible are healthy infants (i.e., free of diseases affecting growth and/ or dietary intake), whose parents are willing to participate in a long-term study and of whom at least one has sufficient knowledge of the German language. Further details about the study have been described elsewhere [6]. The study was approved by the Ethics Committee of the University of Bonn according to the guidelines of the Declaration of Helsinki, and all examinations are performed with parental and later on children’s written consent.

In a previous trend analysis [3], which included the observation periods 1985–2016, we showed that free sugar intake in the DONALD study increased between 1985 and 2005 and decreased thereafter, most pronounced since 2010. Since this last temporal change in the intake of free sugar was observed in 2010 [3], we decided to set the observation period to 2010–2023 for current trend analysis. For the present work, thus, data from all participants aged 3–18 years who completed at least one 3-day weighed dietary record during the years 2010–2023 (4,218 dietary records, *n* = 751) were included. Data from 2023 included all available dietary records (*n* = 174) at the date of analysis in April 2024.

### Data assessment

Dietary intake in the DONALD study is assessed using 3-day weighed dietary records. All foods and beverages consumed by the participant as well as leftovers are weighed and recorded over 3 days by the parents or by older participants themselves, with the use of electronic food scales (± 1 g). Information on recipes (ingredients and preparation) as well as on the types and brands of food items consumed is also requested. This detailed data collection allows estimations of added sugar contents: Based on recipe simulation using labelled ingredients and nutrient contents trained nurses estimate energy and nutrient content, including added sugars, of commercial composite foods, i.e., processed foods and ready-to-eat-meals or snack foods consumed by the DONALD participants. All food items are coded and added to our continuously updated in-house nutrient database LEBTAB [5] together with the nutrient data. Moreover, LEBTAB also contains data on basic foods (e.g., apple or milk), which is based on the German food composition tables BLS 3.02. (https://blsdb.de/).

Total daily energy intake (kcal/d) and daily nutrient intake (%E/d) of the participants, including intake of total sugar (i.e. sum of all monosaccharides and disaccharides in foods and beverages) and added sugar (i.e. sugars that are added to foods or beverages during processing and preparation at home or in manufacture including sugars from honey, syrups and fruit juice concentrates), was then estimated using record and LEBTAB data. Using data on added sugar intake, free sugar intake (%E/d) was also estimated (sum of added sugar intake and intake of sugars from fruits and vegetable juices as well as smoothies). The selection of sugars and its definitions are explained in detail in Perrar et al. 2020 [3].

For the present analysis, anthropometric and socioeconomic characteristics were considered as potentially confounding factors. For anthropometric data, trained nurses conduct standard measurements at each visit to the study centre according to the study protocol [6] of the DONALD study. Parental data for socioeconomic characteristics is collected every 4 years. Details of data assessment [6] and calculation of covariates [3] are described elsewhere.

### Statistical analyses

Statistical analyses were performed using SAS^®^ procedures (version 9.4; Cary, NC, USA). The significance level was set to *p* < 0.05. Results of the descriptive analyses are presented as medians with interquartile range or frequencies and percentages.

As in our previous paper [3] linear, quadratic and cubic age (age^2^, age^3^) and time (time^2^, time^3^) trends in free sugar intake (%E) were analysed using polynomial mixed-effects regression models including both fixed and random effects (PROC MIXED in SAS ^®^). A linear trend reflects a constant increase or decrease in the respective outcome variable over the years or with age. Quadratic and cubic trends indicate that the magnitude of the trend changes over time or with age. Age and time—continuously in years—were the principle fixed effects of the models. The first included record in this examination was considered the baseline time, i.e., time = 0. A repeated statement was considered in the models to account for the lack of independence between repeated measures from the same person. Random effects were considered to allow variation between individuals and families with respect to the initial level (intercept) as well as linear, quadratic and cubic age trends of the respective outcome. The following characteristics were tested as potentially confounding factors: sex (boy/girl), overweight status (yes/no), number of weekdays per 3-day record (1/2/3), maternal overweight (yes/no), high maternal educational status (yes/no), maternal employment (yes/no). Variables that were considered in the final models either modified regression coefficients in the basic models by ≥ 10% or had a significant and independent association with the outcome variable. Furthermore, it was tested whether statistical interactions exist by inserting different interaction terms (age * time, age * sex, time * sex) to the regression model. As there were no interactions, the trends were examined in the entire collective. More details of statistical methods of the trend analyses are described in Perrar et al. 2020 [3].

Since underreporting of food intake can bias the results, a sensitivity analysis excluding underreported records were conducted. Dietary records were considered as underreported when the total energy intake (TEI) was inadequate in relation to the estimated basal metabolic rate (BMR) (according to age- and sex-specific equations of Schofield [7]) using paediatric cut-offs from Sichert-Hellert et al. [8]. This resulted in 452 (10.7%) reords with underreporting.

## Results

In April 2024, 4,218 dietary records from 751 DONALD study participants (46.0% females; 3–18 years; 2010–2023) were available. Per participant, between one (*n* = 144, 19.2%) and fourteen (*n* = 7, 0.9%) dietary records [median (Q1; Q3): 4 (2; 7)] were provided. Participant dietary characteristics stratified by age groups are shown in Table 1. The median total sugar intake was highest for both sexes in 3–5 year olds (girls: 25.6%E, boys: 25.4%E), while median added sugar (girls: 12.0%E, boys: 11.6%E) was highest among 6–10 year olds. Median free sugar intake ranged between 12.2%E among 15–18 year olds and 15.0%E among 6–10 year olds in girls. In boys, median free sugar ranged between 12.9%E among 3–5 year olds and 15.2%E among 6–10 year olds. While girls in toddler age had a slightly higher free sugar intake than boys (13.9%E vs. 12.9%E), boys had higher total and added sugar intakes from 11 to 14 years onwards (Table 1). Stratified by observation years, median free sugar intake ranged between 16.7%E in 2010/2011 and 11.7%E in 2022/2023 (Table 2). Overweight status as well as maternal characteristics reflect the high socioeconomic status of the participants of the DONALD study (Table 2).

**Fig. 1:**
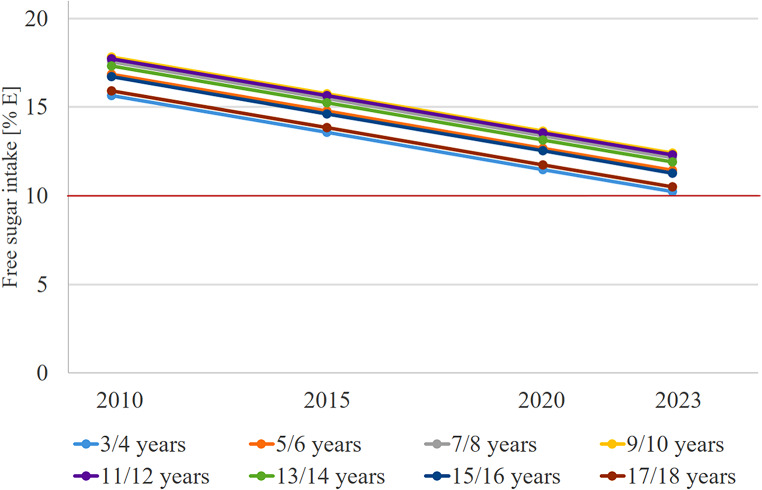
Time and age trends in FS intake of 4,218 dietary records from 751 DONALD study participants (3–18 years) between 2010 and 2023, predicted by polynomial mixed-effects regression models (light blue 3/4 year-olds, orange 5/6 year-olds, grey 7/8 year-olds, yellow 9/10 year-olds, purple 11/12 year-olds, green 13/14 year-olds, dark blue 15/16 year-olds, red 17/18 year-olds). Red line: Maximum recommended intake for free sugar according to the WHO [2]

Free sugar intake changed significantly with age (Table 3; Linear trend: β = 1.3032, *p* < 0.0001; quadratic trend: β = -0.08763, *p* = 0.0079; cubic trend: β = 0.001465, *p* = 0.1574). Figure 1 shows that the youngest (3–4 year-olds) and oldest participants (17–18 year olds) had the lowest intake of free sugar, while adolescents aged 9–10 and 11–12 years had the highest intake. In terms of time trend, the intake of free sugars decreased continuously between 2017 and 2023 (Table 3; β = -0.4175, *p* < 0.0001).

Sensitivity analysis excluding underreported records did not reveal differences in age and time trends from analysis including all records (Table S1).

**Table 1:** Dietary characteristics from 1940 records from 360 female and 2278 records from 391 male DONALD study participants (3–18 years) between 2010 and 2023, stratified by age groups

**Girls**				
	**3–5 years**	**6–10 years**	**11–14 years**	**15–18 years**
**n**_**records**_	399	616	469	456
**n**_**participants**_	182	192	169	170
**age**	4.0 (3.1, 5.0)	8.1 (7.0, 9.1)	12.3 (11.3, 13.4)	16.6 (15.3, 17.7)
**TEI [kcal]**	1116 (990, 1254)	1498 (1306, 1690)	1734 (1513, 1991)	1753 (1499, 2025)
**Protein intake [%E/d]**	12.9 (11.5, 14.2)	12.6 (11.4, 14.1)	12.8 (11.5, 14.3)	13.6 (12.0, 15.2)
**Fat intake [%E/d]**	33.4 (29.8, 37.7)	33.8 (29.8, 37.5)	34.7 (30.2, 38.1)	35.0 (31.1, 38.7)
**Carbohydrates [%E/d]**	52.3 (48.3, 56.2)	52.5 (48.4, 56.7)	51.3 (47.6, 55.7)	50.4 (46.5, 53.7)
**Total sugar [%E/d]**	25.6 (20.6, 30.6)	24.1 (20.0, 29.1)	23.0 (19.0, 28.5)	21.0 (16.6, 26.2)
**Free sugar intake [%E/d]**	13.9 (9.1, 18.5)	15.0 (11.3, 19.4)	14.5 (10.7, 19.4)	12.2 (8.5, 16.9)
**Added sugar [%E/d]**	10.0 (7.2, 13.0)	11.6 (9.0, 14.8)	11.2 (8.5, 15.3)	9.7 (6.5, 13.3)
**Boys**				
	**3–5 years**	**6–10 years**	**11–14 years**	**15–18 years**
**n**_**records**_	464	755	561	498
**n**_**participants**_^**1**^	213	225	199	175
**age**	4.0 (3.1, 5.0)	8.1 (7.0, 9.1)	12.3 (11.4, 13.3)	16.3 (15.4, 17.4)
**TEI [kcal]**	1194 (1040, 1374)	1652 (1435, 1870)	1940 (1670, 2204)	2319 (2009, 2750)
**Protein intake [%E/d]**	13.0 (11.7, 14.4)	12.9 (11.4, 14.2)	13.4 (11.8, 15.0)	14.0 (12.6, 16.1)
**Fat intake [%E/d]**	32.8 (28.9, 36.6)	34.2 (30.6, 37.6)	34.8 (30.9, 38.9)	34.8 (30.8, 38.9)
**Carbohydrates [%E/d]**	53.2 (49.0, 56.9)	52.1 (48.2, 55.7)	50.4 (46.8, 54.6)	49.1 (45.0, 53.1)
**Total sugar [%E/d]**	25.4 (21.3, 29.7)	24.3 (20.1, 28.8)	22.4 (18.6, 27.0)	20.8 (16.2, 25.5)
**Free sugar intake [%E/d]**	12.9 (9.0, 17.5)	15.1 (11.5, 19.5)	14.7 (11.1, 18.6)	13.3 (9.1, 17.8)
**Added sugar [%E/d]**	9.6 (6.6, 13.5)	12.0 (9.0, 15.3)	12.0 (8.6, 15.3)	11.0 (7.2, 14.6)

Values are medians (25th, 75th percentile)

TEI total energy intake; E = Percentage of total daily energy intake

^1^Since participants are asked to complete a record each year, more than one dietary records per participant are included in the analyses

**Table 2:** Dietary characteristics from 4,218 dietary records from 751 DONALD study participants (3–18 years) between 2010 and 2023, stratified by years

	Total sample						
	2010/2011	2012/2013	2014/2015	2016/2017	2018/2019	2020/2021	2022/2023
**n**_**records**_	768	722	702	639	558	494	335^1^
**n**_**participants**_^**2**^	449	406	408	379	328	302	239
**Females**	371 (48.3)	343 (47.5)	324 (46.2)	290 (45.4)	251 (45.0)	211 (42.7)	150 (44.8)
**Age**	9.1 (6.0, 13.5)	10.0 (6.1, 14.0)	10.3 (6.2, 14.2)	10.2 (6.1, 14.1)	11.0 (6.4, 14.9)	10.7 (7.0, 15.0)	10.0 (5.5, 14.0)
**Dietary variables**							
TEI (kcal/d)	1616 (1317, 1945)	1633 (1322, 1992)	1643 (1294, 1995)	1619 (1331, 1984)	1646 (1337, 2019)	1623 (1335, 2003)	1545 (1253, 1978)
Protein (%E/d)	13.1 (11.6, 14.4)	13.2 (11.9, 14.9)	13.1 (11.8, 14.6)	13.1 (11.6, 14.7)	13.2 (11.7, 14.8)	13.1 (11.6, 14.8)	12.7 (11.3, 14.1)
Fat (%E/d)	33.3 (29.9, 37.0)	33.3 (29.5, 37.2)	33.8 (29.9, 38.3)	34.2 (30.4, 37.9)	35.0 (30.9, 38.9)	35.2 (31.4, 38.9)	35.0 (31.2, 38.9)
Carbohydrates (%E/d)	52.0 (48.5, 56.1)	52.1 (48.1, 56.2)	51.7 (47.3, 55.7)	51.2 (47.6, 55.5)	50.4 (46.7, 55.1)	50.1 (46.5, 54.6)	50.8 (47.4, 55.3)
Total sugar (%E/d)	26.0 (21.5, 31.1)	24.4 (19.8, 29.6)	23.4 (19.6, 27.4)	23.3 (19.4, 28.3)	22.3 (18.2, 26.6)	21.0 (17.1, 25.3)	21.0 (16.6, 24.6)
Free sugar (%E/d)	16.7 (12.3, 21.3)	15.5 (11.0, 19.9)	14.0 (10.8, 18.6)	14.4 (10.7, 18.3)	13.3 (9.2, 17.1)	11.7 (7.8, 15.3)	11.7 (7.8, 15.0)
Added sugar (%E/d)	12.0 (8.9, 15.9)	11.7 (8.6, 15.4)	11.2 (8.1, 14.4)	11.2 (8.0, 14.7)	10.8 (7.4, 13.8)	9.6 (6.6, 12.9)	9.9 (6.9, 13.0)
**Underreporting [n**_**records**_ **(%)]** ^3^	60 (7.8)	85 (11.8)	79 (11.3)	67 (10.5)	58 (10.4)	61 (12.4)	42 (12.5)
**Anthropometrics**							
BMI	16.9 (15.5, 19.8)	16.8, 15.3, 19.8)	17.0 (15.4, 19.7)	16.7 (15.5, 19.9)	17.1 (15.5, 19.7)	17.3 (15.5, 19.7)	16.6 (15.4, 19.7)
Overweight [n_**records**_ (%)]^4^	100 (13.0)	90 (12.5)	91 (12.1)	81 (12.6)	66 (11.8)	71 (14.4)	48 (14.3)
**Maternal characteristics** [n_**records**_ (%)]^5^							
Overweight^6^	291 (38.1)	270 (37.7)	257 (37.0)	241 (38.4)	202 (37.8)	170 (36.4)	85 (28.3)
High educational status^7^	621 (80.9)	611 (84.7)	611 (87.3)	578 (90.5)	520 (93.2)	459 (92.9)	312 (93.1)
Employment	625 (81.4)	613 (84.9)	607 (86.5)	570 (89.2)	526 (94.3)	470 (95.1)	321 (95.8)

Values are medians (25th, 75th percentile) or frequencies (%)

*TEI* total energy intake; *%E* percentage of energy intake

^1^ included all available dietary records at the date of analysis (April 2024), which are not all collected records from 2023 due to time difference in coding

^2^Since participants are asked to complete a record each year, more than one dietary records per participant are included in the analyses

^3^ Number of underreported records. Pediatric cutoff values according to Sichert-Hellert et al., 1998 [8]

^4^ Number of records, which were collected from overweight children and adolescents. International age and sex specific BMI cut-off values for children and adolescents for overweight according to Cole et al., 2007 [9]

^5^ Maternal characteristics during dietary record collection

^6^ BMI > 25 kg/m²

^7^ ≥12 years of schooling

**Table 3:** Time and age trends in FS intake of 4,218 dietary records from 751 DONALD study participants (3–18 years) between 2010 and 2023

	Age trend per year of age (3–18 years)^a^			Time trend per study year (1910–2023)^b^		
	Age β (*p*)	Age² β (*p*)	Age³ β (*p*)	Time β (*p*)	Time² β (*p*)	Time³ β (*p*)
**Free sugar** ^**c**^ Unadjusted model Fully adjusted model	1.3600 (< 0.0001) 1.3032 (< 0.0001)	-0.09634 (0.0034) -0.08763 (0.0079)	0.001744 (0.0918) 0.001465 (0.1574)	-0.4168 (< 0.0001) -0.4175 (< 0.0001)	- -	- -

Age and time trends were tested using polynomial mixed-effects regression models

^a^Age=linear age trend, age²=quadratic age trend, age³=cubic age trend

^b^time=linear time trend, time²=quadratic time trend, time³=cubic time trend

^c^Model contains a random statement for the family level with an unstructured covariance structure and a random statement for the person level with an unstructured covariance structure. Adjusted for number of weekdays per record (1/2/3) and overweight status (yes/no)

## Discussion

The present study provides an update on current sugar intake as well as age and time trends of intake of free sugar of children and adolescents in Germany. Even if the median free sugar intake is still above the recommendations, our results show a decline in free sugar intake between 2010 and 2023 among children and adolescents. In a previous publication from the DONALD study, which showed trends in the intake of total, added and free sugar from 1985 to 2016 also among 3–18 year olds [3], free sugar intake decreases since 2005, most notably since 2010. The present analyses indicate that this decreasing trend continued.

A possible reason for the observed decreasing trend in free sugar intake could be an increased awareness of parents and participants themselves on the consequences of high sugar intake or specific sugary foods e.g. sugar sweetened beverages. The high sugar intake of children and adolescents has been a common topic in politics [1] and therefore also in media for many years. Since December 2018, the German government has been pursuing a national reduction and innovation strategy for sugar, fats and salt intake [1]. This includes voluntary reformulation measures to reduce sugar in ready to eat products, so far except sweets as well as a ban on added sugar in children’s teas has also been passed. However, in DONALD we saw a decline in sugar intake already before 2018, pointing towards potential different reasons for the decline. It is furthermore questionable if the DONALD participants habitually consumed already reformulated products or if high-sugar food groups such as sweets, sugar-sweetened beverages and juices were specifically avoided. Previous analyses have shown that the decrease in free sugar intake between 2010 and 2016 is due to a decline in the intake of specific food groups [10]. In particular, the intake of free sugars from sugar-sweetened beverages and fruit juices decreased, while the decline in the intake of free sugars from sweets was less pronounced.

It should be considered critically, that dietary data is self-reported in the present analyses, since the underestimation of habitual energy intake is a common known bias in observational studies [8]. In addition, non-plausible dietary records showed a markedly lower intake level of added sugar than plausible records [8]. Although our sensitivity analysis does not support the notion of bias from general underreporting, we cannot preclude the possibility that the observed decline in free sugar intake partly reflects nutrient specific misreporting. The observed trend in our previous analyses [3] was confirmed using predictive biomarkers for total sugar intake [4]. Replicating the present analyses using biomarker data would help rule out specific underreporting of high-sugary foods.

Regardless of the ongoing decline in free sugar intake since 2010, median intake in this German sample still exceeds the WHO recommendation of a maximum of 10%E free sugar per day [2] in all age groups and in all years of observation. Descriptively, there seems also a stagnation in the median free sugar intake between 2020/2021 and 2022/2023. However, this stagnation cannot be confirmed based on the regression analyses. Therefore, our data indicate that the intake of free sugars among children and adolescents should continue to be monitored. Particular attention should be paid to critical ages, as in the present evaluation the median intake among 6- to 14-year-olds over the past 13 years is quite high at around 15%E. In addition, the available trends show that these age groups are most vulnerable to a high sugar intake: The age trend in free sugar intake between 2010 and 2023 is similar to the trend in 1985–2016 [3]. In both, the intake of free sugars per day increases continuously up to early adolescence and then decreases again. Although the differences between the intakes during primary school age and early adolescence are small. While in previous analyses the oldest participants showed the lowest intake of free sugars [3], in 2010–2023 it was the youngest (3/4 years) and oldest participants (17/18 years). This indicates an age and time interaction i.e. that the strength of the decline in free sugar intake varied depending on the age of the participants, which however could not be confirmed statistically in the current trend analyses.

The high sugar intake observed among children and adolescents supports the current sugar reduction measures of the German ministry. Until 2025, sugar reduction of at least 20% in breakfast cereals for children as well as of 15% each in sweetened dairy products for children and in soft drinks and fruit drinks with added sugar are envisaged [1]. Since previous data show, that the main free sugar source of children are sweets [10], it should be discussed whether the envisaged measures are sufficient to reduce intake to below 10% E/d or whether this food group should also be included in future measures. Especially, as the intake of free sugar could even be higher in representative samples.

A limitation of the present evaluation is the relatively high socioeconomic status of the DONALD participants, due to the comprehensive study design, limiting the generalizability of our observations to the general paediatric population in Germany [6]. To the best of our knowledge, representative data on the intake of free sugars among children and adolescents in Germany, which also includes families with a rather low socio-economic status, has not yet been published. It is possible that the intake of free sugars decreases less in this population group. However, socio-economic factors i.e. maternal educational status as well as employment status were taken into account as potential covariates in the present analysis. In contrast to our previous trend analyses [3], socioeconomic factors were not identified as confounders in the present analysis. Relevant confounding factors were number of weekdays per record and overweight status of the participants only. Both factors were already relevant in the earlier trend analysis on free sugar intake [3].

There are also several strengths to the current study and analysis that should be mentioned. The main strength of the DONALD study is its longitudinal design, allowing time and age trend analyses covering large time periods as well as the entire childhood and adolescence. The 3-day weighed dietary records and the continuously updated in-house nutrient database LEBTAB [5] allow the estimation of total, added and free sugar intake. Hence, the DONALD study is one of few studies, which can calculate data on free sugar intake. In addition, LEBTAB accounts for changes in recipes over time. If the recipe of a product was changed e.g. due to reformulation measures, a new entry was added to LEBTAB, whilst the entry for the product with the old recipe was marked, but retained in the database for dietary analyses. This consideration of recipe changes by industrial manufacturers in our nutrient database enables time trend analyses in dietary intake such as the present one.

In conclusion, free sugar intake continues to decline among children and adolescents in Germany, but still exceeds the WHO recommendation. Regardless of the observation years, children between 6 and 14 years of age appear to be particularly vulnerable to a high intake of free sugar. Our data support the relevance of the current political discussions on reducing sugar intake. Further measures, including the extension of reformulation measures to other food groups, the introduction of taxes on sugar sweetened beverages or the introduction of labels, which include sugar content limits, should be discussed.

## Electronic supplementary material

Below is the link to the electronic supplementary material.

Supplementary Material 1

## Data Availability

Data of the DONALD study is available upon request to epi@uni-bonn.de.
