# Supplementary material for: Intake of free sugar among children and adolescents in Germany declines – current results of the DONALD study
Source: Eur J Nutr. 2024 Jul 5;63(7):2827–33. doi: 10.1007/s00394-024-03456-1 (PMC11490435; doi:10.1007/s00394-024-03456-1)
Supplement: Supplementary file 1 — Supplementary Material 1 [file 394_2024_3456_MOESM1_ESM.docx]

**Supplementary Information**

**Table S1:** Time and age trends in FS intake of DONALD study participants (3–18 years) between 2010 and 2023, excluding underreported records (n=452)

|  | **Age trend per year of age (3-18 years)^a^** | | | **Time trend per study year (1910-2023)^b^** | | |
| --- | --- | --- | --- | --- | --- | --- |
|  | **Age**  **β (p)** | **Age²**  **β (p)** | **Age³**  **β (p)** | **Time**  **β (p)** | **Time²**  **β (p)** | **Time³**  **β (p)** |
|  |  |  |  |  |  |  |
| **Free sugar^c^**  Unadjusted model  Fully adjusted model | 1.2166 (0.0003)  1.1809 (0.0004) | -0.07993 (0.0209)  -0.07438 (0.0316) | 0.001327 (0.2272)  0.001153 (0.2939) | -0.4282 (<0.0001)  -0.4292 (<0.0001) | -  - | -  - |
|  |  |  |  |  |  |  |

Age and time trends were tested using polynomial mixed-effects regression models

^a^Age=linear age trend, age²=quadratic age trend, age³=cubic age trend

^b^time=linear time trend, time²=quadratic time trend, time³=cubic time trend

^c^Model contains a random statement for the family level with an unstructured covariance structure and a random statement for the person level with an unstructured covariance structure. Adjusted for number of weekdays per record (1/2/3) and overweight status (yes/no).
